# *Research Integrity and Peer Review*—past highlights and future directions

**DOI:** 10.1186/s41073-018-0047-1

**Published:** 2018-03-06

**Authors:** Stephanie L. Boughton, Maria K. Kowalczuk, Joerg J. Meerpohl, Elizabeth Wager, Elizabeth C. Moylan

**Affiliations:** 1 Springer Nature, 4 Crinan Street, London, N1 9XW UK; 2 Institute for Evidence in Medicine (for Cochrane Germany Foundation), Medical Center – University of Freiburg, Freiburg, Germany; 3 SideView, Princes Risborough, Buckinghamshire UK; 4 BMC (Springer Nature), 4 Crinan Street, London, N1 9XW UK

## Abstract

In May 2016, we launched *Research Integrity and Peer Review*, an international, open access journal with fully open peer review (reviewers are identified on their reports and named reports are published alongside the article) to provide a home for research on research and publication ethics, research reporting, and research on peer review. As the journal enters its third year, we reflect on recent events and highlights for the journal and explore how the journal is faring in terms of gender and diversity in peer review. We also share the particular interests of our Editors-in-Chief regarding models of peer review, reporting quality, common research integrity issues that arise during the publishing process, and how people interact with the published literature. We continue to encourage further research into peer review, research and publication ethics and research reporting, as we believe that all new initiatives should be evidence-based. We also remain open to constructive discussions of the developments in the field that offer new solutions.

## Introduction

These are exciting times for *Research Integrity and Peer Review*. Since launching in May 2016 [1], the journal has published a range of articles on diverse topics spanning broad themes within publication ethics, research reporting and peer review. Our four Editors-in-Chief have been on the road speaking at conferences and events including the World Conference on Research Integrity [2] and the International Congress on Peer Review and Scientific Publishing [3], a UK parliamentary inquiry into research integrity [4] and the Global Evidence Summit [5]. The past year has also been a year of change for the journal, as we have said goodbye (and thank you) to one of our founding editors, Iveta Simera, and hello (and welcome) to Joerg Meerpohl as the Editor-in-Chief of the journal’s research reporting section. Here, we share with you some of the most memorable moments from the past 18 months.

## It's all about the research

An analysis of the articles published up to January 2018 (Table 1) reveals that the majority of articles fall within the research and publication ethics section, and perhaps this is to be expected given the section’s broad scope and the funding for research in this area. Published articles have covered issues that can occur during the phases of research and publication. These include how research is funded [6] and reviewed [7, 8], guidelines on research integrity [9], researchers’ perceptions on research misbehaviour [10] and their views on the publishing process [11]. Challenging topics have been tackled also, including how to handle appropriate disclosure of conflicts of interest [12], how to agree authorship contributions [13] and what is appropriate in terms of citation practices [14, 15] and text-recycling [16, 17]. Of course, potential concerns within publication ethics do not cease with publication and we have published articles that investigate the incidence of plagiarism [18], reasons for retractions [19] and the varied uses of expressions of concern [20].

**Table 1:** Article topics published within the areas of research and publication ethics, research reporting and peer review

Journal section	Subject area (reference number in brackets)
Research and publication ethics	Conflicts of interest disclosure [12]
	Guidelines on research integrity [9]
	Costs of ethical review [7]
	Citation bias [14, 15]
	Plagiarism [18]
	Research misbehaviours [10]
	Text recycling [16, 17]
	Reasons for retractions [19]
	Uses of expressions of concern [20]
	Research ethics review [8]
	Research funding [6]
	Author contributions [13]
	Author perceptions of publishing [11]
Research reporting	Reporting on sex and gender [24, 25]
	Standards of reporting [21–23]
	Data sharing [29, 30]
	Trial registration [27, 28]
	Readers perceptions on research [31]
	Factors associated with online media attention of research articles [32]
Peer review	Training in peer review [34, 35]
	Reviewer recruitment [37, 38]
	Mentoring in peer review [36]
	Views on peer review models [39]
	Peer review of grant proposals [33]
Of general relevance	Proceedings of the 4th World Conference on Research Integrity [42]
	Proceedings from the IV Brazilian Meeting on Research Integrity, Science and Publication Ethics [43]

Within the reporting section of the journal, initiatives that aim to increase the transparency and reproducibility of research have been published. These include research on standards of reporting [21] including how to correctly identify the most relevant reporting guidelines [22] and how to ensure they are up to date [23]. Of particular importance is the recognition that accurate reporting of sex and gender is key for research across multiple disciplines [24, 25], and the guidelines have been endorsed by EQUATOR [26]. Other initiatives which have been discussed include investigations of the extent of trial registration and the publication of clinical trials [27, 28] and the need for more collaborative efforts to facilitate and incentivise appropriate data-sharing [29, 30]. We can also learn about the factors that affect readers’ perception of research, and those which influence the media attention given to articles post-publication from research in these areas [31, 32].

In the peer review section, several articles published have focused on diverse aspects of the peer review process. These span a deeper dive into how grant review panels work [33] and the need for support for peer reviewers, particular in areas of training [34, 35] and mentoring [36]. Other topics include a study of reviewer recruitment in the field of ecology [37, 38] and research into the views of junior hospital doctors on their understanding of models of peer review [39]. It is great to see the journal is sharing experience of research into peer review from varied subject areas and connecting diverse communities, which was one of our aims in launching a multi-disciplinary journal.

Of course, the topics of the articles ultimately accepted for publication in the journal do not reflect the range of submissions we have seen. While we welcome all research within the areas of publication ethics, research reporting and peer review, we would particularly like to encourage submissions on the use of alternative metrics; research into different models of peer review; studies exploring the role of gender and diversity in peer review and how institutions work on supporting completeness, accuracy and transparency in research reporting and preventing and handling research misconduct.

## Gender equality at *Research Integrity and Peer Review*

Given recent interest into gender and diversity in peer review [40] and inspired by investigations at other journals [41], we wanted to find out how the journal is faring in terms of gender and diversity (with respect to its editorial board, authors and peer reviewers). Of course, the journal is still growing, so the sample size is small, but we hope this will be a useful benchmark to monitor changes in future.

In most cases, it was possible to determine gender of individuals simply by name recognition, but in some cases, we had to check other sources for further information. The gender balance among our Editors-in-Chief is positively skewed in favour of females (3:1), but across the editorial board, the ratio is more even with 18 females (40%) and 27 males (60%). However, perhaps the gender composition of the board may reflect the composition of the research field as a whole? This may be the case given that of 33 articles published so far, 12 articles (36%) have female corresponding authors while 21 articles (63%) have male corresponding authors. In terms of peer reviewers, gender diversity is more balanced with 31 (45%) women and 38 (55%) men.

We were also able to investigate the geographic location of editorial board members, authors and peer reviewers who had agreed to review as one measure of ‘diversity’. The diversity among the Editors-in-Chief is biased in favour of Europe. However, the rest of the editorial board (comprising 45 individuals) span locations across the following continents: Europe (17, 38%), North America (13, 29%), South America (2, 4%), Africa (2, 4%), Asia (6, 13%) and Oceania (5, 11%), although there is a bias towards Europe and North America.

In terms of the location of corresponding authors, of 33 articles published up to January 2018, excluding conference proceedings [42, 43], we see a narrower range of diversity with the majority of authors located in North America (14, 42%), Europe (12, 36%) and Oceania (7, 21%).

In terms of 69 reviewers who supplied reviews for published articles, the diversity is somewhat broader with reviewers located across Europe (38, 55%), North America (25, 36%), Africa (1, 1%), Asia (1, 1%) and Oceania (4, 6%).

We are proud of the gender and diversity balance we are currently achieving across our editorial board and our reviewers, and we will continue to work to ensure appropriate balance in terms of gender and diversity in the future. While it is difficult to directly influence gender and diversity imbalances in submissions to the journal, we welcome submissions from across the world, especially Asia, Africa and South America.

## Editors-in-Chief on the road

Our Editors-in-Chief attended key events in the area of research integrity [2, 4], peer review [3] and evidence-based policy [5] during 2017. The 5th World Conference on Research Integrity (WCRI), held in May 2017 in Amsterdam, focussed on transparency and accountability, themes which are very much interlinked at the journal through the open peer review model that the journal has adopted. Liz Wager (who works as an independent freelance consultant and trainer) played an active role in the conference programme. She championed what journals can do to improve transparency in research reporting [44] and also reviewed what countries can do to better support research integrity [45], a timely topic in the UK at the moment given the government inquiry into research integrity at which Liz also gave oral evidence [46]. Liz also led a discussion on how editors can cooperate and liaise with research institutions [47].

The WCRI was followed in September 2017 by the 8th International Congress on Peer Review and Scientific Publication, held in Chicago. The congress aims to encourage research into all aspects of peer review and scientific publication and establish the evidence-base on which researchers can improve the conduct, reporting and dissemination of research. The specific theme for 2017 was ‘enhancing the quality and credibility of science’ which very much corresponds with the scope of *Research Integrity and Peer Review* although the journal scope spans all research, including the humanities and social sciences*.* Stephanie Boughton, Maria Kowalczuk and Liz Wager were able to share their own recent research at the conference.

Stephanie presented an insight into the types of queries raised with BMC’s Research Integrity Group, showing that ‘ethics and consent’ and ‘data issues’ were the main themes, and two thirds of queries were raised by editors ahead of publication [48]. Maria compared journals operating on open, single-blind or double-blind peer review models, showing that while more invitations on average were sent to secure reviewers for the open model, the difference with other models was not insurmountable [49]. Liz’s analysis of PubPeer comments on articles in leading medical journals revealed that the frequency of comments requiring journal action was low, and although editors were unaware of the methodological issues raised in that way, they often followed up independently [50].

Also in September 2017, the global evidence-based health care community got together in Cape Town, South Africa, at the first ever Global Evidence Summit (GS), co-hosted by Cochrane, Guidelines International Network, Campbell Collaboration, Joanna Briggs Institute and the International Society for Evidence-Based Health Care (ISEHC). While the scope of the GES was broad focusing on evidence production as well as evidence use, e.g. in guidelines, reporting quality of trials and systematic reviews was discussed in various sessions such as ‘Reporting evidence synthesis’, ‘Improving conduct and reporting of evidence synthesis’, or ‘Tools to communicate and use evidence’. Joerg co-organized a special session on dissemination bias in qualitative research, and how this might impact on qualitative evidence syntheses [51], an area where further research is clearly needed.

## Future directions on research integrity and peer review?

Looking back, it is encouraging to see the wealth of research being conducted in areas of research integrity, reporting and peer review, some of which we were able to publish in *Research Integrity and Peer Review*. This year, we look forward to hearing more about discussions and investigations that are being shared by the ASAPbio community into transparency, recognition and innovation in peer review in the life sciences ([52], the PEERE International Conference on Peer Review [53] and the PRINTEGER (Promoting Integrity as an Integral Dimension of Excellence in Research) conference [54].

Of course, some initiatives will help, others may fail, and that is fine as long as research in these areas is data-driven. In our role as co-Editors-in-Chief of *Research Integrity and Peer Revie*w we would love to hear about your research and how it could make a difference. Thank you to our authors and our reviewers, our Editorial Board and our readers for all their continued support. We look forward to working with you in 2018, and beyond.
